# Identification of miRNA expression profile in middle ear cholesteatoma using small RNA-sequencing

**DOI:** 10.1186/s12920-024-01932-5

**Published:** 2024-06-18

**Authors:** Mengyao Xie, Qi Tang, Shu Wang, Xiaowu Huang, Zhiyuan Wu, Zhijin Han, Chen Li, Bin Wang, Yingying Shang, Hua Yang

**Affiliations:** 1 Department of Otolaryngology, Peking Union Medical College Hospital, Chinese Academy of Medical Sciences and Peking Union Medical College, No.1 Shuaifuyuan, Beijing, 100730 P.R. China; 2 Chinese Academy of Medical Sciences and Peking Union Medical College, Beijing, 100730 P.R. China; 3 Department of Otolaryngology, Shenzhen Hospital, Southern Medical University, Shenzhen, 518100 P.R. China

**Keywords:** MicroRNA, Cholesteatoma, Small RNA sequencing, Functional enrichment

## Abstract

**Background:**

The present study aims to identify the differential miRNA expression profile in middle ear cholesteatoma and explore their potential roles in its pathogenesis.

**Methods:**

Cholesteatoma and matched normal retroauricular skin tissue samples were collected from patients diagnosed with acquired middle ear cholesteatoma. The miRNA expression profiling was performed using small RNA sequencing, which further validated by quantitative real-time PCR (qRT-PCR). Target genes of differentially expressed miRNAs in cholesteatoma were predicted. The interaction network of 5 most significantly differentially expressed miRNAs was visualized using Cytoscape. Further Gene Ontology (GO) and Kyoto Encyclopedia of Genes and Genome (KEGG) pathway enrichment analyses were processed to investigate the biological functions of miRNAs in cholesteatoma.

**Results:**

The miRNA expression profile revealed 121 significantly differentially expressed miRNAs in cholesteatoma compared to normal skin tissues, with 56 upregulated and 65 downregulated. GO and KEGG pathway enrichment analyses suggested their significant roles in the pathogenesis of cholesteatoma. The interaction network of the the 2 most upregulated (hsa-miR-21-5p and hsa-miR-142-5p) and 3 most downregulated (hsa-miR-508-3p, hsa-miR-509-3p and hsa-miR-211-5p) miRNAs identified TGFBR2, MBNL1, and NFAT5 as potential key target genes in middle ear cholesteatoma.

**Conclusions:**

This study provides a comprehensive miRNA expression profile in middle ear cholesteatoma, which may aid in identifying therapeutic targets for its management.

## Background

Cholesteatoma is a benign lesion with abnormal proliferation of keratinizing stratified squamous epithelium in the middle ear. Although benign, it is often characterized by local invasive growth in the temporal bone which may lead to diverse complications including hearing loss, vestibular dysfunctions and facial never palsy. Moreover, life-threatening complications can occur with the aggressive intracranial extension of cholesteatoma. So far, surgical removal is considered the only curative approach for cholesteatoma. However, high residual risks and recurrence rate after surgery have been observed. It is reported that the residual rate of cholesteatoma is around 5-27.8% [1–4] and the recurrence rate can range from 7.6–34.9% [3, 5]. Many patients with cholesteatoma undergo reoperation or even multiple operations due to its recidivism, resulting in diminished quality of life and heavy economic burden. Thus, there is a clear need for investigating the underlying mechanisms and developing novel therapeutic strategies of cholesteatoma.

Despite numerus studies on the pathogenesis of cholesteatoma, the mechanisms are still not fully elucidated. There have been four major theories that were widely recognized for acquired cholesteatoma: (1) retraction pocket theory, (2) immigration theory, (3) squamous metaplasia theory, and (4) papillary ingrowth theory [6]. Many scholars believed that the etiopathogenesis of acquired cholesteatoma could not be completely explained by one single theory. The biological behaviors of abnormal proliferation and bone erosion which are similar to malignant tumors involves a complex pathophysiological process. With recent advances, the current understanding of acquired cholesteatoma pathogenesis has deepened into the microscopic level of cells and molecules. Genetics and epigenetic factors have been taken into consideration in the formation and progress of cholesteatoma. Several research suggested the genetic associations of cholesteatoma in syndromic and familial forms [7, 8]. Also, researchers have recently paid attention to the role of non-coding RNAs in cholesteatoma pathogenesis which have certain therapeutic potential [9, 10]. Complete removal of the lesion, preservation of normal middle ear mucosa, and restoration of the middle ear ventilation and drainage system during surgery help reduce the recurrence of cholesteatoma [11–13]. On this basis, we attempt to explore additional auxiliary conservative methods that can work synergistically to enhance treatment effectiveness and further reduce recurrence of cholesteatoma.

MicroRNA (miRNAs) is a type of non-coding RNA which plays a key role in epigenetic regulations. It consists of approximately 18 to 25 nucleotides and has high sequence conservation [14]. Based on its regulative effect on its target mRNA, miRNAs have been suggested as potential therapeutic targets in tumors and chronic inflammatory diseases [15, 16]. Quite a few miRNAs have been demonstrated to be significantly differentially expressed in cholesteatoma tissue compared with normal tissues. For instance, high expression levels of miR-21 and miR-let-7a were found in cholesteatoma tissue. However, they exert opposing functions. MiR-21 promotes proliferation and invasion of keratinocytes in cholesteatoma and inhibits apoptosis process [17, 18]. A balancing mechanism was proposed that they might have the joint regulation of cholesteatoma growth and invasiveness [19]. To investigate miRNAs and their roles in cholesteatoma, it is necessary to obtain the full miRNA expression profile. RNA-sequencing (RNA-seq) technique is a sequencing-based technique which can directly detect RNA sequences with high sensitivity and accuracy. Different from microarray, it can identify new RNA sequences.

Here, we collected samples from patients with acquired retraction pocket cholesteatoma. We used small RNA-seq to present miRNA expression profile of acquired cholesteatoma and compare with matched normal skin tissue. The sequencing results were further validated via quantitative real-time polymerase chain reaction (qRT-PCR). Our present study predicted and analyzed target genes of differentially expressed miRNAs, aiming to identify epigenic alterations and potential therapeutic targets for cholesteatoma.

## Methods

### Patients and specimens

Middle ear cholesteatoma tissue and matched normal retroauricular skin tissue specimens were obtained from the same patient. A total of 6 patients(3 males and 3 females)aged between 35 and 75 years were enrolled who underwent surgery for middle ear cholesteatoma between October 2021 and April 2022 at the department of Otorhinolaryngology, Peking Union Medical College Hospital. All participants provided written informed consent before enrollment. Ethical approval was obtained through the Ethics Committee of Peking Union Medical College Hospital (no. JS- 2726). All specimens were stored at -80℃ immediately after being excised until further RNA extraction.

### RNA extraction and RNA quantitation

Total RNA was isolated from the cholesteatoma and skin tissue using TRIzol® Reagent (Invitrogen) according to the manufacturer’s protocol. DNase I RNase-free (TaKara) was used to remove genomic DNA. We further monitored RNA degradation and contamination on 1% agarose gels. Then, RNA concentration was tested using ND-2000 (NanoDrop Technologies). Finally, RNA integrity was assessed using a 2100 Bioanalyzer (Agilent Technologies, Santa Clara, CA, USA). Only high-quality RNA sample (OD260/280 = 1.8 ~ 2.2, OD260/230 ≥ 2.0, RIN ≥ 7, 28 S:18 S ≥ 1.0, > 3 µg) was used as input material for the sequencing library.

### Small RNA sequencing

Sequencing libraries were established using NEBNext® Multiplex Small RNA Library Prep Set for Illumina® (NEB, USA.). After cDNA synthesis, polymerase chain reaction (PCR) amplification was performed using LongAmp Taq 2X Master Mix, SR Primer.

for illumina and index (X) primer. Then PCR products were purified and 140–160 bp(the length of small noncoding RNA plus the 3’ and 5’ adaptors)target DNA fragments were obtained. The quality assessment of the libraries was performed on the Agilent Bioanalyzer 2100 system with DNA High Sensitivity Chips. Qualified libraries were sequenced on an Illumina platform and 50 bp single-end reads were generated.

To ensure the accuracy of further bioinformatic analysis, raw paired end reads were trimmed and filtered by fastx toolkit software (http://hannonlab.cshl.edu/fastx_toolkit/). Through the BLAST search of the miRbase (http://www.mirbase.org/), the perfectly matched sequences were used to count and analyze the known miRNAs. In addition, novel miRNAs can be predict with the use of the characteristics of the hairpin structure of miRNA precursors. The available software miRDeep2 was to predict novel miRNAs.

The expression level of each miRNA was calculated according to the transcripts per million reads (TPM) method. MiRNAs with |log2 fold change (FC)| > 1 and P value < 0.05 were considered to be significant differently expressed between cholesteatoma tissue and matched skin tissue.

### Quantitative real-time PCR (qRT-PCR) validation

Total RNA was extracted using TRIzol® Reagent (Invitrogen). After assessment of RNA quality and integrity, RNA was reverse transcribed to synthesize complementary deoxyribonucleic acid (cDNA) using the Hifair ® III first Strand cDNA Synthesis SuperMix kit (Yeasen, China). Then, six differentially expressed miRNAs obtained from sequencing data were validated via qRT-PCR using Hieff® qPCR SYBR Green Master Mix (Yeasen, China). U6 was used as an internal control and primer sequences are listed in Table 1. The comparative cycle threshold method (2^−ΔΔCq^) was used to compare the relative expression levels of each miRNA. P-value < 0.05 was considered to be statistically significant.

**Table 1:** Primer sequence of RT-qPCR

miRNAs	Primers
hsa-miR-21-5p-RT	GTCGTATCCAGTGCAGGGTCCGAGGTATTCGCACTGGATACGACTCAACATC
hsa-miR-21-5p-F	TCAGACTGATGTTGAGTCGTATCC
hsa-miR-451a-RT	GTCGTATCCAGTGCAGGGTCCGAGGTATTCGCACTGGATACGACAACTCAGT
hsa-miR-451a-F	CCGTTACCATTACTGAGTTGTCG
hsa-miR-142-5p-RT	GTCGTATCCAGTGCAGGGTCCGAGGTATTCGCACTGGATACGACAGTAGTGC
hsa-miR-142-5p-F	TAGAAAGCACTACTGTCGTATCCA
hsa-miR-200b-3p-RT	GTCGTATCCAGTGCAGGGTCCGAGGTATTCGCACTGGATACGACTCATCATT
hsa-miR-200b-3p-F	TGCCTGGTAATGATGAGTCGT
hsa-miR-211-5p-RT	GTCGTATCCAGTGCAGGGTCCGAGGTATTCGCACTGGATACGACAGGCGAAG
hsa-miR-211-5p-F	CCCTTTGTCATCCTTCGCCT
hsa-miR-508-3p-RT	GTCGTATCCAGTGCAGGGTCCGAGGTATTCGCACTGGATACGACTCTACTCC
hsa-miR-508-3p-F	AGCCTTTTGGAGTAGAGTCGT
U6-F	CGGCAGCACATATACTAAAATTGGA
U6-R(rt)	ATTTGCGTGTCATCCTTGCG
Universal reverse primer	CAGTGCAGGGTCCGAGGTAT

### Prediction of miRNA target genes and functional enrichment analysis

Target genes of differentially expressed miRNAs were predicted using TargetScan (www.targetscan.org/) and miRanda (http://mirtoolsgallery.tech/mirtoolsgallery/node/1055). In addition, the regulatory network of the 5 most significantly expressed miRNAs (hsa-miR-21-5p and hsa-miR-142-5p, hsa-miR-508-3p, hsa-miR-509-3p and hsa-miR-211-5p) and their target genes were visualized using Cytoscape (http://www.cytoscape.org/). Furthermore, Gene Ontology (GO) annotation was obtained from GO database (http://www.geneontology.org/). GO and Kyoto Encyclopedia of Genes and Genomes (KEGG) pathway enrichment analysis were performed using Goatools (https://github.com/tanghaibao/GOatools) and KOBAS (http://kobas.cbi.pku.edu.cn/home.do) respectively. P-values were calculated using Fisher exact test. Bonferroni-corrected P-value<0.05 was considered to be statistically significant.

### Statistical analysis

Statistical analysis (except for RNA-seq data analysis and bioinformatic analysis described above) was performed using GraphPad Prism 8.0 software (https://www.graphpad.com/scientificsoftware/prism/). Data were reported as means ± SE. After verification of normal distribution, measurement data were analyzed with the Student’s t-test to compare the differences of miRNA expression between the two groups. P-value < 0.05 was considered to be statistically significant.

## Results

### Differentially expressed miRNAs between cholesteatoma and matched normal skin

**Fig. 1:**
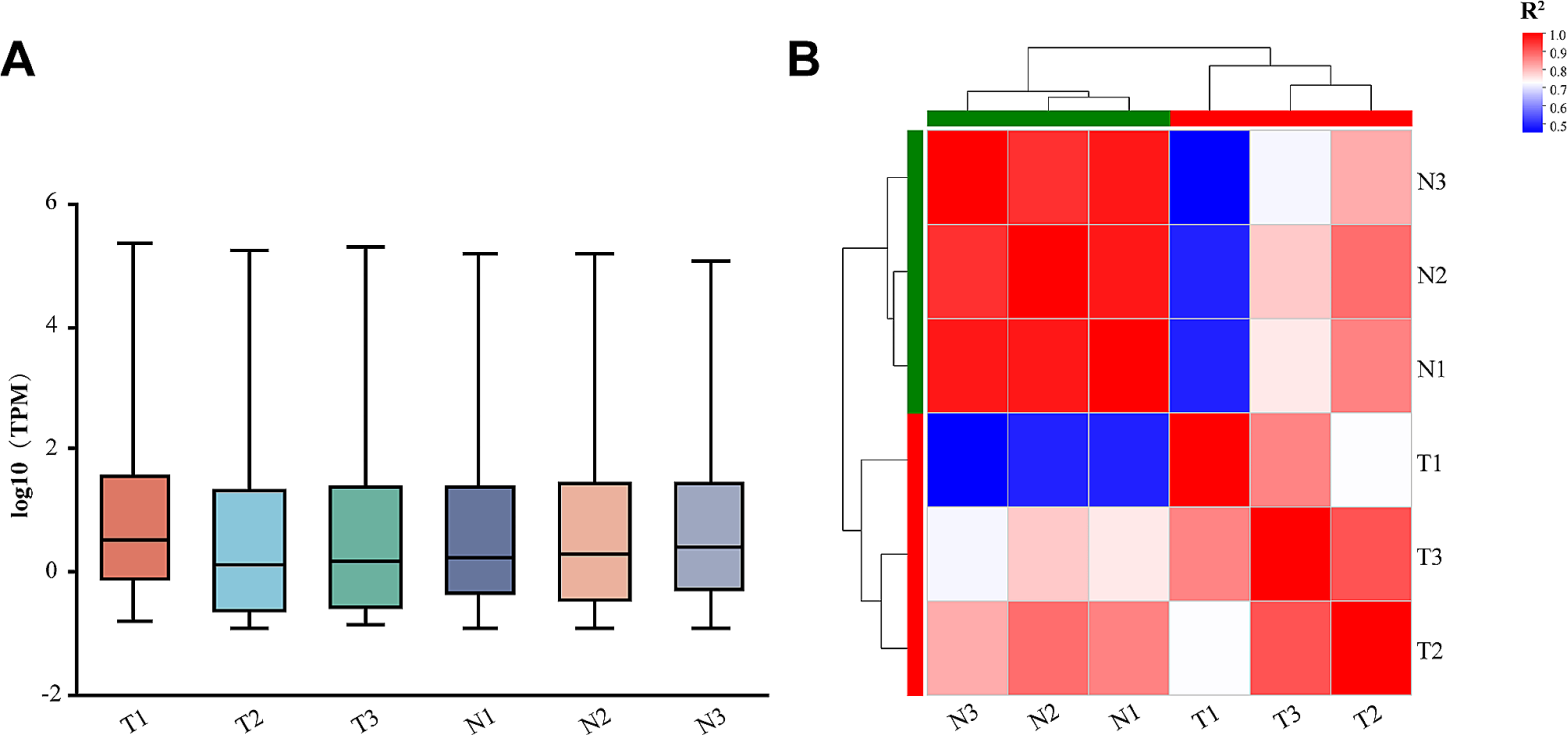
miRNA expression analysis of cholesteatoma samples and matched skin samples. (**A**) Box plot showing the distribution of miRNA expression in each sample after normalization. The abscissa is the sample name, and the ordinate is log10(TPM). Horizontal lines indicate the median. (**B**) Correlation analysis among each sample. The color of the blocks represents R-square value of Spearman’s correlation. The greater the value is, the higher the correlation is. (T1-T3: cholesteatoma samples; N1-N3: matched skin samples)

**Fig. 2:**
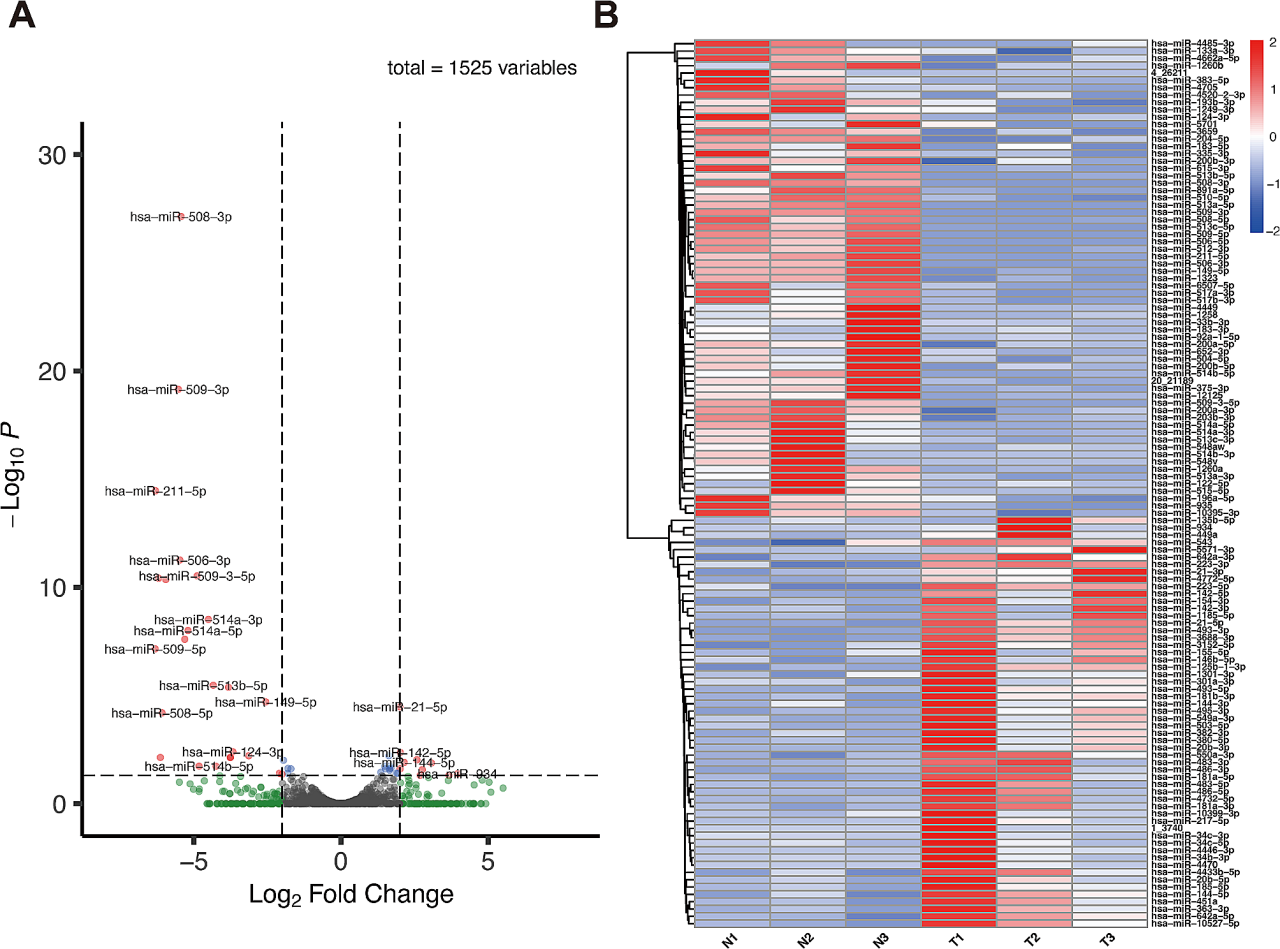
Differentially expressed miRNAs between cholesteatoma and matched normal skin. (**A**) Enhanced volcano plot of differentially expressed miRNAs. The dashed horizontal line shows significance level (*P* = 0.05), and the dashed vertical lines indicate |log2 (Fold Change) |= 1. Red dots represent significantly upregulated and downregulated miRNAs. (**B**) Hierarchical clustering of differentially expressed miRNAs. Red color represents high expression level and blue color represents low expression level

MiRNA sequencing was performed to examine the miRNA expression in cholesteatoma and matched skin tissue. The box plot showed miRNA expression distributions in each sample after normalization (Fig. 1A). We further conducted a correlation analysis to test for biologically repeated correlations between samples. A high degree of correlations was achieved among samples of each group, while the correlation between samples from the two groups was quite low (Fig. 1B). 1525 miRNAs were detected, of which 1430 were known miRNAs and 95 were novel miRNAs. Then, we compared differentially expressed miRNAs between two groups. A total of 121 significantly differentially expressed miRNAs were identified with the threshold of fold change > 2.0 or fold change < 0.5 and P value < 0.05, which consists of 56 upregulated and 65 downregulated miRNAs. An enhanced volcano plot visualized differentially expressed miRNAs based on the threshold values (Fig. 2A). The hierarchical clustering was also presented to illustrate the differential miRNA expression profiles (Fig. 2B).

### qRT-PCR verification of differentially expressed miRNAs

**Fig. 3:**
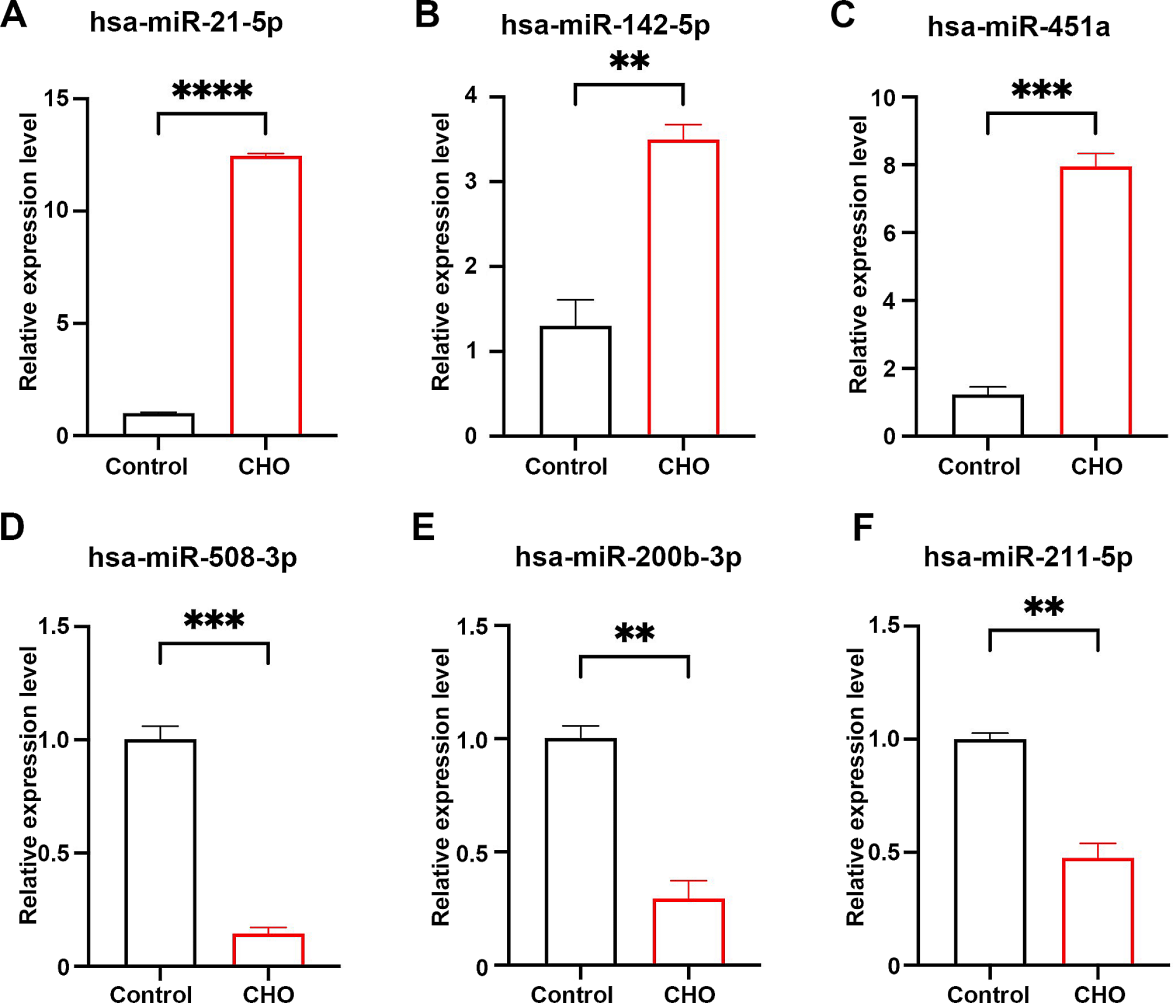
qRT-PCR verification of the relative miRNAs expression levels between cholesteatoma (CHO) and normal skin (Control). (**A**) hsa-miR-21-5p; (**B**) hsa-miR-142-5p; (**C**) hsa-miR-451a; (**D**) hsa-miR-508-3p; (**E**) hsa-miR-200b-3p; (**F**) hsa-miR-211-5p. T-test, with **P < 0.01, t-test, with ***P < 0.001; t-test, with ****P < 0.0001

To validate the miRNA sequencing results, we selected 6 miRNAs (hsa-miR-21-5p, hsa-miR-142-5p, hsa-miR-451a, hsa-miR-508-3p, hsa-miR-200b-3p, hsa-miR-211-5p) from 121 significantly differentially expressed miRNAs to detect their expression levels in cholesteatoma and normal skin using qRT-PCR (Fig. 3). The results displayed significantly higher expression levels of hsa-miR-21-5p (*P*<0.0001), hsa-miR-142-5p (*P* = 0.0034) and hsa-miR-451a (*P* = 0.0001) in cholesteatoma tissue. The expression of hsa-miR-508-3p (*P* = 0.0002), hsa-miR-200b-3p (*P* = 0.0017) and hsa-miR-211-5p (*P* = 0.0016) was reduced in cholesteatoma compared with skin tissue. The expression trends of these miRNAs were consistent with our miRNA sequencing results.

### Functional enrichment and pathway analysis of differentially expressed miRNAs target genes

**Fig. 4:**
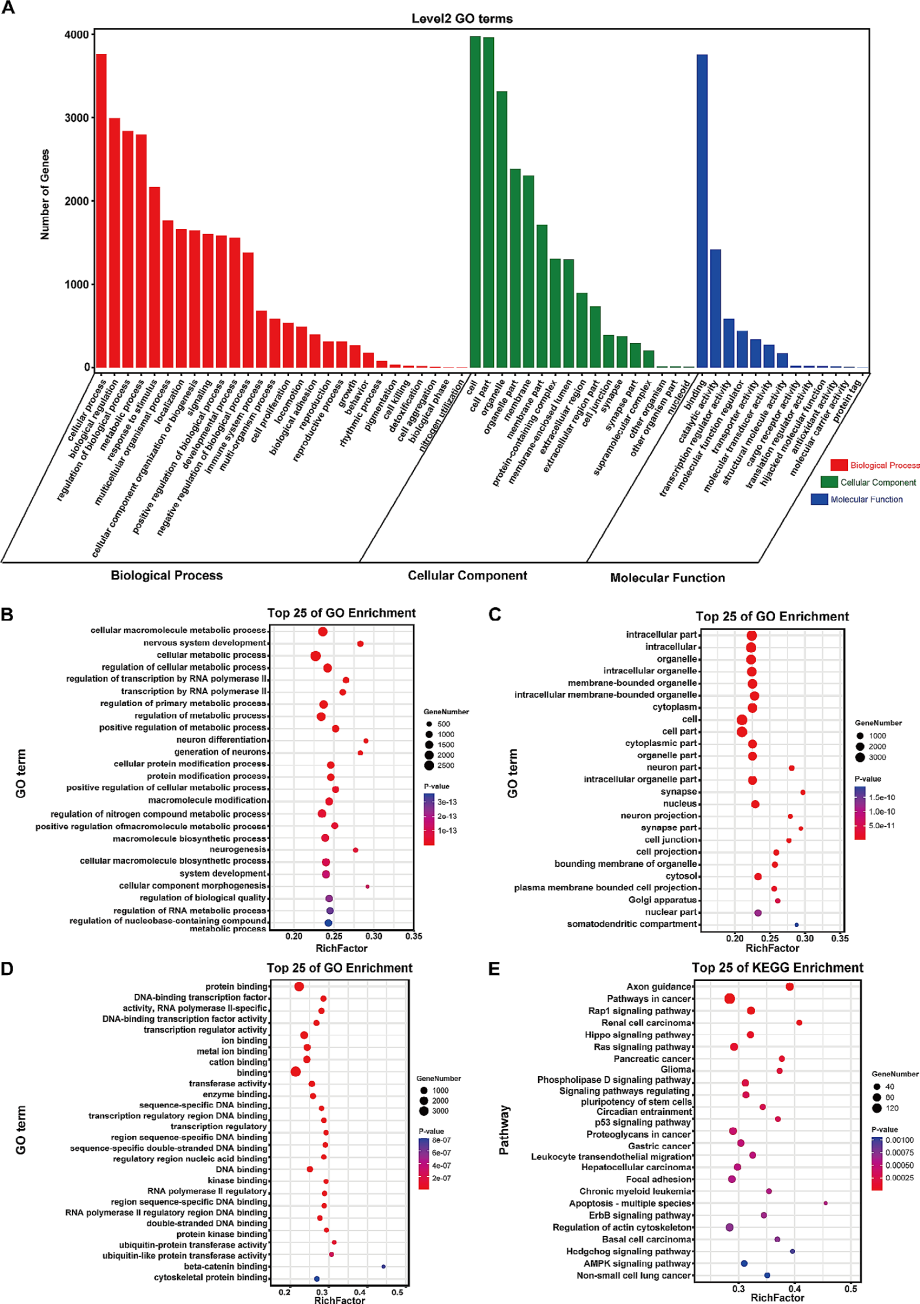
GO and KEGG analysis of differentially expressed miRNAs target genes. (**A**) GO secondary annotation classification of differentially expressed miRNAs target genes. The abscissa shows the level 2 GO terms, and the ordinate represents the numbers of genes in the GO term. (**B-D**) GO functional enrichment analysis of differentially expressed miRNAs target genes. (**B**) The top 25 enriched GO terms in BP; (**C**) The top 25 enriched GO terms in CC; (**D**) The top 25 enriched GO terms in MF. (**E**) The top 25 enriched KEGG pathways of differentially expressed miRNAs target genes. The abscissa displays GO terms or pathways, and the ordinate shows the rich factor which represents the degree of enrichment. The larger value of rich factor indicates greater degree of enrichment. The color of the bubble represents the P-value, and the size of the bubble indicates the gene number in the GO term or pathways

Potential target genes of 121 differentially expressed miRNAs in cholesteatoma were predicted using TargetScan and miRanda. We next investigated their possible functions in the pathogenesis of cholesteatoma via GO analysis. A total of 58 Level 2 GO terms were obtained in GO secondary classification (Fig. 4A). All the target genes annotated were classified into three categories: biological process (BP), cellular component (CC), and molecular function (MF). Further GO enrichment analysis was performed and the top 25 enriched GO terms in each category were listed (Fig. 4B-D). In BP, a large number of target genes were enriched in cellular metabolic process, protein modification process, indicating their potential roles in onset and progression of cholesteatoma. As for CC, high enrichment in intracellular, organelle, cytoplasm and cell were noted. Enrichment for MF revealed that the majority of target genes involved in protein binding, DNA binding and transcription regulator activity. Moreover, pathway enrichment analysis was conducted based on KEGG database and the results were mapped out in the bubble plot (Fig. 4E). The top 3 highest enrichment pathways were axon guidance, pathways in cancer and Rap1 signaling pathway. It’s noteworthy that target genes were also significantly enriched in pathways like Hippo, Ras, p53, AMPK pathway.

### Interaction network of 5 most significantly differentially expressed miRNAs target genes

**Fig. 5:**
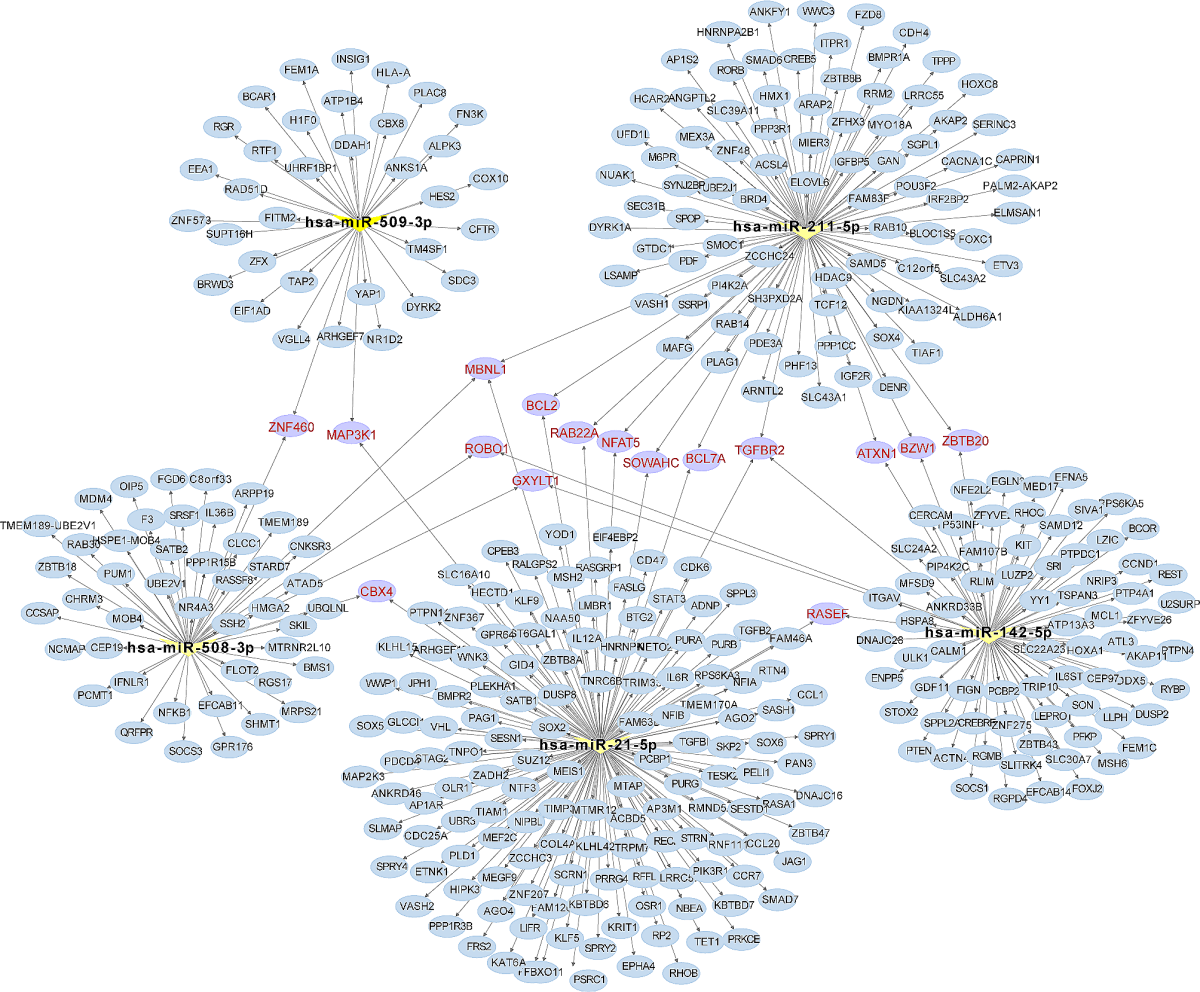
Interaction network of 5 most significantly differentially expressed miRNAs target genes. Yellow triangle nodes represent miRNAs. Circular nodes represent target genes. Common target genes are marked in red

To further identify genes that are most likely regulated by miRNA in cholesteatoma, we chose 2 most upregulated (hsa-miR-21-5p and hsa-miR-142-5p) and 3 most downregulated (hsa-miR-508-3p, hsa-miR-509-3p and hsa-miR-211-5p) differentially expressed miRNAs to map out their target genes and visualize their interaction relationship by Cytoscape (Fig. 5). The interaction network was composed of 5 miRNA nodes and 389 target gene nodes. Interestingly, we found that hsa-miR-21-5p, hsa-miR-142-5p and hsa-miR-211-5p all targeted TGFBR2 and hsa-miR-508-3p, hsa-miR-211-5p and hsa-miR-21-5p all regulated MBNL1 expression. Moreover, the common target genes of hsa-miR-211-5p and hsa-miR-21-5p were NFAT5, BCL2, BCL7A, RAB22A and SOWAHC. Other common targeted relationships between miRNA and genes were manifested in the network. These common target genes mainly participated in the process of cell proliferation, angiogenesis and immune response, which relates to the possible pathogenesis of cholesteatoma. Further studies are required to confirm the findings of differentially expressed miRNAs and their target genes.

## Discussion

Middle ear cholesteatoma is a relatively common disease in otolaryngology [20], the pathogenesis and mechanisms of which have been drawing growing attention. Numerous studies revealed the roles of miRNAs in the formation and progression of cholesteatoma [9, 10].In the present study, we identified 121 significantly differentially expressed miRNAs in acquired retraction pocket cholesteatoma tissues via small RNA sequencing. Among them, 56 miRNAs were upregulated, and 65 miRNAs were downregulated. The differentially expressed miRNAs play crucial roles in various processes within cholesteatoma, including the regulation of metabolic processes, transcriptional control, and more. We further explored the co-regulated genes of 5 most significantly differentially expressed miRNA, which might have potential associations with the mechanism of cholesteatoma.

Previous studies have demonstrated the involvement of miR-21 in the development and progression of cholesteatoma. Friedland et al. were the first to discover the function of miR-21 in cholesteatoma formation. They found out that the upregulation of miR-21 expression in cholesteatoma was negatively correlated with the expression of PTEN and PDCD4. They proposed that hsa-miR-21 target PTEN and PDCD4 genes, which inhibits keratinocytes apoptosis and promotes their proliferation, invasion, and migration [18]. The results were also confirmed by subsequent studies [17, 19]. Chen et al. validated the expression level of miR-21 and assessed PTEN and PDCD proteins expression among adult and pediatric cholesteatoma patients. They also explored miR-21 functions at cellular level with the transfection of miR-21 mimics and inhibitors into cholesteatoma keratinocytes. The results verified that miR-21 promotes the proliferation and invasion of cholesteatoma keratinocytes. Our present study showed that hsa-miR-21-5p, as one of the miR-21 family, was significantly upregulated in cholesteatoma tissues with 4.017-fold higher than in normal skin tissues. The result was consistent with the previous research. High expression level of miR-let-7a was found in some studies, which affects the expression and function of miR-21 and counterbalances the excessive keratinocytes proliferation in cholesteatoma [19, 21]. However, hsa-miR-let-7a was not found to be expressed differentially between cholesteatoma and normal skin in the present study. Our sequencing data was consistent with one microarray study [22], but in contrast with the above two research. Therefore, additional studies into the relationship between miR-let-7a expression and cholesteatoma are needed.

Other significantly differentially expressed miRNAs we found in this study have been reported to participate in the pathogenesis of several diseases. For instance, hsa-miR-509-3p suppresses invasion, metastasis and epithelial-mesenchymal transition in cancers like hepatocellular carcinoma [23] and melanoma [24]. Partial epithelial-mesenchymal transition (p-EMT) was also observed in acquired middle ear cholesteatoma and congenital cholesteatoma by Takahashi et al [25]. We propose that the downregulation of miR-509-3p shown in the sequencing data have possible association with the p-EMT process in cholesteatoma, which merits further exploration. Hsa-miR-211-5p was proved to promote osteogenic differentiation of human-derived mesenchymal stem cell [26]. The expression of hsa-miR-211-5p was found to be diminished in cholesteatoma tissues. The finding might explain for the bone destruction mechanism of cholesteatoma. Few studies investigated the potential roles of these differentially expressed miRNAs in the cholesteatoma. Moreover, the regulatory network of miRNAs is extremely complicated. One single miRNA can target multiple genes and a specific gene can be regulated by multiple miRNAs. Hence, identification of the downstream gene targets of specific miRNAs is essential to the understanding of the role miRNAs play in the cholesteatoma. In the current study, the interaction network of 5 most significantly differentially expressed miRNAs target genes was visualized to investigate the key target genes and to indicate directions for further mechanistic research.

By constructing the interaction network, we had several interesting findings. The network generated by Cytoscape revealed that the top 5 most significantly differentially expressed miRNAs share some target genes. Hsa-miR-21-5p, hsa-miR-142-5p and hsa-miR-211-5p all targeted transforming growth factor beta receptors II (TGFBR2). TGFBR2 gene encodes TGF-β receptor II (TGFβRII) protein, which serves as a significant part of the TGF-β signaling pathway. TGF-β ligand binds to TGFβRII on the cell membrane and form a heterotrimeric complex with TGF-β receptor II (TGFβRI) to phosphorylate downstream mediators in the cytoplasm [27]. TGF-β has been confirmed to stimulate fibroblasts proliferation and differentiation in pulmonary and myocardial fibrosis [28, 29]. Additionally, the depletion of TGFβRII from CD4 + T cells reprogram the tumor vasculature and triggers cancer cell death, which in turn inhibits cancer progression in mice [30]. Combined with the results of this study, we suppose that TGFBR2 could be a promising target in the drug development strategies of cholesteatoma. Moreover, a recent study showed that suppression of TGF-β signaling enables long-term proliferation of stem/progenitor cells of the tympanic membrane and the middle ear mucosa epithelium [31]. The finding also provides a theoretical basis for the clinical application of the treatment strategy mentioned above. In addition, hsa-miR-508-3p, hsa-miR-211-5p and hsa-miR-21-5p all regulated muscleblind-like protein 1 (MBNL1) expression. MBNL1 participated in the regulation of alternative RNA splicing in tumor growth and metastasis [32]. Other common target genes are also noteworthy. NFAT5, one of the co-regulated genes of hsa-miR-211-5p and hsa-miR-21-5p, can modulate the immune function of T cells and macrophages [33]. The degree of ossicular erosion in cholesteatoma was proved to be associated with the macrophage activation phenotypes according to an immunohistochemical study [34]. To our knowledge, the role of above-mentioned target genes in cholesteatoma has not been investigated, which deserves further research.

There are some limitations to the present study. The sample size was relatively small. Further analysis with larger sample is needed. Also, more detailed exploration is required to elucidate the function of candidate miRNAs in the mechanism and pathogenesis of cholesteatoma. Furthermore, we predicted the target genes and the enriched pathways of the differentially expressed miRNAs. The experimental verification was lacking. We intend to pursue this in the subsequent research to find possible therapeutic targets for the management of cholesteatoma.

## Conclusions

In conclusion, we identified a comprehensive miRNA expression profile of acquired middle ear cholesteatoma. A total of 121 significantly differentially expressed miRNAs were found in cholesteatoma compared to normal skin, among which 56 were upregulated and 65 were downregulated. Differentially expressed miRNAs of cholesteatoma mainly involved in cellular metabolic processes, cell proliferation, transcription regulator activity and immune response. The interaction network analysis of the two most upregulated miRNAs (hsa-miR-21-5p and hsa-miR-142-5p) and the three most downregulated miRNAs (hsa-miR-508-3p, hsa-miR-509-3p, and hsa-miR-211-5p) identified TGFBR2, MBNL1, and NFAT5 as potential key target genes in middle ear cholesteatoma.

## Data Availability

The datasets generated during the current study are available from the corresponding author on reasonable request.

## Abbreviations

AMPK: Adenosine monophosphate-activated protein kinase
BCL2: B-cell lymphoma 2
BCL7A: B-cell lymphoma 7 protein family member A
BP: Biological process
CC: Cellular component
GO: Gene Ontology
KEGG: Kyoto Encyclopedia of Genes and Genome
MBNL1: Muscleblind-like protein 1
MF: Molecular function
miRNA: microRNA
NFAT5: Nuclear factor of activated T cells
PDCD4: Programmed cell death 4
p-EMT: Partial epithelial-mesenchymal transition
PTEN: Phosphatase and tensin homolog deleted on chromosome ten
qRT-PCR: Quantitative real-time polymerase chain reaction
RAB22A: Ras-related protein Rab-22 A
RNA-seq: RNA-sequencing
SOWAHC: Sosondowah ankyrin repeat domain family member C
TGFBR2: Transforming growth factor beta receptors II
